# Evaluation of ARFI elastography for detecting active mastitis in sheep with previous fibrous lesions: a study of mammary parenchyma and supramammary lymph nodes

**DOI:** 10.1590/1984-3143-AR2023-0160

**Published:** 2024-09-23

**Authors:** Yuri da Silva Bonacin, Victor José Correia Santos, Marjury Cristina Maronezi, Luiz Paulo Nogueira Aires, Michele Pereira Machado, Beatriz Longo Barbosa, André Marcos Santana, Priscila Del’Aguila-Silva, Paulo Aléscio Canola, Marcus Antônio Rossi Feliciano, José Antônio Marques

**Affiliations:** 1 Faculdade de Ciências Agrárias e Veterinárias, Universidade Estadual Paulista “Júlio de Mesquita Filho”, Departamento de Clínica e Cirurgia Veterinária, Jaboticabal, SP, Brasil; 2 Faculdade de Ciências Agrárias e Veterinárias, Universidade Estadual Paulista “Júlio de Mesquita Filho”, Departamento de Patologia, Reprodução e Saúde Única, Jaboticabal, SP, Brasil; 3 Centro Universitário Moura Lacerda - Campus Unit, Ribeirão Preto, SP, Brasil; 4 Universidade Estadual de Maringá, Departamento de Medicina Veterinária, Maringá, PR, Brasil; 5 Faculdade de Zootecnia e Engenharia de Alimentos, Universidade de São Paulo, Departamento de Medicina Veterinária, Pirassununga, SP, Brasil

**Keywords:** Inflammation, milk, sheep, ultrasound

## Abstract

The aim of the study was to evaluate the use of Acustic Radiation Force Impulse (ARFI) elastography in mammary parenchyma and supramammary lymph nodes, for detection of active mastitis in sheep with naturally infected chronic fibrous lesions. 27 female sheep were included and B-mode ultrasound and ARFI elastography images were obtained, acquiring qualitative (echogenicity and echotexture) and quantitative (shear rate, depth and short/long axis ratio) variables of 48 mammary glands. The glands were divided into three experimental groups: control group (CG) - healthy animals; LSCC- animals that presented fibrous lesions and SCC (somatic cell count) less than 500 x 10^3^ cls/mL; HSCC: animals that presented fibrous lesions and SCC (somatic cell count) more than 500 x 10^3^ cls/mL; The qualitative variables using B-mode ultrasonography, including echotexture and echogenicity, showed no significant differences between the evaluated groups and tissues (p = 0.9336 and p = 0.233, respectively) .In healthy areas of the gland, it was an increasing in shear wave velocity (SWV) in LSCC than in HSCC (p=0.04). When comparing the fibrosis in the LSCC and HSCC groups with their respective normal areas, the velocity increased in both groups: LSCC (p= 0,0007) and HSCC (p= 0,0001). When comparing the areas of fibrosis in LSCC and HSCC with the CG parenchyma, there was an increase in LSCC (p=0.001) and HSCC (p=0.0001). B-mode ultrasound indicate predominance of hypoechoic echogenicity in lymph nodes and reduced short/long axis ratio in cases of active subclinical mastitis. The supramammary lymph node showed increased SWV when comparing the CG with HSCC groups (p=0.02) and GC with LSCC (p=0.04). B-mode ultrasonography is useful for evaluating the mammary parenchyma, however, its application as a standalone diagnostic technique is not recommended. ARFI elastography indicates potential cutoff points for differentiating subclinical mastitis from healed mastitis, highlighting its importance as a tool for distinguishing normal areas from fibrous parenchymal areas. While this study did not establish specific cutoff points due to sample size limitations, further research with larger sample sizes could explore and define these critical thresholds.

## Introduction

Mastitis is described as an inflammation of the mammary parenchyma, of multifactorial etiology (Menzies and Ramanoon, 2001). The evolution of mastitis in sheep was summarized by Quinlivan (1968) in four levels, currently used according to its clinical presentation. One can further differentiate infectious mastitis into clinical and subclinical, where the latter has no evident clinical symptoms, and can cause a reduction in milk production and changes in milk quality, mainly by an increase in the number of somatic cells (defense cells and mammary gland desquamation cells), acting as a silent disease in the herd (Knuth et al., 2019; Puggioni et al., 2020).Mastitis can lead to other negative impacts, such as neonatal mortality, reduced weight gain of lactating lambs (Arsenault et al., 2008).

Numerous diagnostic methods can be used to detect mastitis. B-mode ultrasound is an important tool in the identification of clinical and subclinical mammary gland changes, where it is feasible to visualize and evaluate the mammary gland parenchyma integrity, mammary gland cistern, teat cistern or even the local blood supply in the search for morphological changes (Hussein et al., 2015; Barbagianni et al., 2017;). The supramamammary lymph nodes can also be evaluated (Barbagianni et al., 2017). In a study with cows, Bradley et al. (2001) reported increased lymph node dimensions in animals with high somatic cell counts, which can also be observed in sheep females (Hussein et al., 2015).

ARFI elastography (Acoustic Radiation Force Impulse) is an advanced imaging technique used for evaluating tissue stiffness. It enables a qualitative and quantitative assessment and offers real-time evaluations (Nightingale, 2011). Elastography examination finds extensive application in human medicine for distinguishing between malignant and benign breast tumours (Zhou et al., 2016), as well as for discriminating idiopathic granulomatous mastitis from mammary neoplasms, contributing effectively to clinical practice (Sousaris and Barr, 2016; Toprak et al., 2022; Yağcı et al., 2017). In the field of veterinary medicine, elastography examination demonstrates a high level of specificity (97.2%) and sensitivity (94.7%) in distinguishing between malignant from benign mammary neoplasms in dogs (Feliciano et al., 2017).

The early detection of mastitis holds paramount significance due to the limitations and time-consuming nature of traditional methods like somatic cell counts and bacterial isolation, which are expensive and provide results over several days. In this context, ultrasound serves as an effective adjunctive tool (Hussein et al., 2015), since chronic mastitis induces notable alterations in the mammary parenchyma, including different levels of fibrosis (Quinlivan, 1968).

Considering the aforementioned circumstances, the present study aims to investigate the sonographic findings obtained through B-mode ultrasonography and ARFI elastography in cases of active mastitis in female sheep. The main objective of the study is to investigate if ultrasound imaging techniques can be used as additional diagnostic tools to differentiate between two groups of ewes: one group with chronic udder lesions from past mastitis cases but low somatic cell counts, and another group with ongoing subclinical mastitis and similar udder lesions.

## Methods

### Animals

The study was approved by the Ethics Committee on Animal Use (CEUA) under protocol number 017013/18. Twenty-seven female sheep, from the cross between the Dorper and Santa Inês breeds, aged 6±2 years, at the 60th day of lactation, were used. The animals came from a private breeding farm, with a reported high rate of mastitis, kept in a semi-intensive regime and with their respective lactating lambs.

The animals were categorized into three distinct experimental groups based on the results obtained from somatic cell counts and the presence or absence of fibrotic lesions within the mammary parenchyma. The Control Group (CG) consisted of healthy female subjects devoid of fibrotic lesions and exhibiting somatic cell counts below 500 x 10^3^ cls/mL. The second group, designated as the Fibrotic Lesions with Low Somatic Cell Count Group (LSCC), encompassed animals displaying fibrotic lesions alongside somatic cell counts lower than 500 x 10^3^ cls/mL. Lastly, the Fibrotic Lesions with High Somatic Cell Count Group (HSCC) consisted of animals showcasing fibrotic lesions in addition to somatic cell counts exceeding 500 x 10^3^ cls/mL.

### Milk sample collection

The animals were manually restrained and maintained in a stable position throughout the procedure. Following restraint, the teats were meticulously cleaned using Povidine Iodine Topical spray solution. Subsequently, the teats were dried using disposable paper towels, and manual milking was performed. During the milking process, the initial three milk jets were discarded, after which a representative sample was collected by measuring 10 mL of milk from each half of the udder. The milk samples were carefully transferred to sterile 50 mL vials containing bronopol preservative, with two vials allocated per animal. The resulting 48 vials were appropriately labelled and promptly dispatched to the laboratory for further analysis.

### Somatic cell Count (SCC)

The milk samples were sent to the Laboratory of the Milk Clinic, located in the Department of Animal Science of the University of São Paulo (Esalq/USP), Piracicaba campus, São Paulo, Brazil. Cell count was performed by the automatic method with an electronic infrared counter Bentley 2000® (Bentley Instruments, Chaska, United States), a widely adopted technique for bovine cell counting.

### B-mode ultrasonography

The animals were housed in two brick stalls with access to hay and water ad libitum, and subsequently, they were transported in pairs to the ultrasonography room. Manual restraint was employed, maintaining them in a "sitting dog" position, with the gluteal region supported by a foam cushion and the forelimbs elevated above the ground. Ultrasonography was conducted using ACUSON S2000 equipment (SIEMENS Healthineears, Munich, Germany), equipped with a multifrequency (9.0 to 13.0 MHz) linear transducer. All sonographic evaluations were carried out by a single skilled operator. Ultrasonographic assessment of the mammary gland aimed to identify fibrous lesions within the right and left halves of the mammary parenchyma. In cases where fibrotic lesions were detected, the following qualitative variables were examined: echotexture (homogeneous or heterogeneous) and echogenicity (hypoechogenic, hyperechogenic, or mixed). The same set of variables was also observed within the unaffected mammary gland parenchyma for subsequent comparative analysis.

At the completion of the mammary parenchyma scan, the supramammary lymph nodes associated with each mammary half were identified for qualitative evaluation. The assessment included the observation of echogenicity, echotexture, and shape (elongated or rounded). Furthermore, a quantitative evaluation was conducted, encompassing measurements of the long and short axes of the lymph nodes.

### ARFI elastography

Following the B-mode evaluation, ARFI elastography was conducted utilizing the Virtual Touch Tissue Quantification™ software (Siemens, Germany) on the breast parenchyma and supramammary lymph nodes. At the designated areas of interest, including the healthy breast parenchyma, breast parenchyma with fibrosis, and supramammary lymph nodes, three quantitative measurements of local Shear Wave Velocity (SWV) (m/s) and depth (cm) were obtained using calliper positioning, as outlined by Simões et al. (2018). Simultaneously, during the examination, qualitative variables pertaining to both tissues were meticulously observed, including deformation capacity (soft or hard) and pattern (homogeneous or heterogeneous). The identical procedure was executed for both halves of the breast.

### Statistical analysis

The statistical software R® (R Foundation for Statistical Computing, Vienna, Austria) was utilized for the tests and the confidence interval used was 95% for all tests performed. The data underwent the Kolmogorov-Smirnov normality test and the parametric variable SCC was log transformed, and then an analysis of variance (ANOVA) test followed by post-hoc Tukey-test. The remaining variables did not require transformation. Supramammary lymph node shear velocity was compared using ANOVA with a post hoc Dunnett test, assuming that CG serves as the control group. A paired t-test was employed to compare shear wave velocity (SWV) between the normal parenchyma of each group and the fibrous portion in the LSCC and HSCC groups. Other variables were subjected to ANOVA and post hoc Tukey tests. Pearson's correlation was used to analyse the qualitative variables of B-mode ultrasound and elastography.

## Results

**Figure 1:**
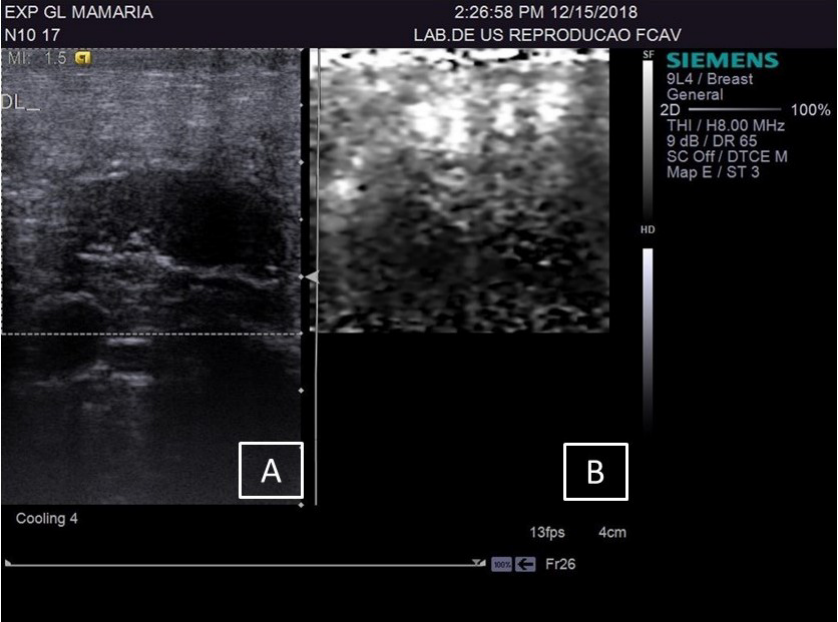
(A) B-mode ultrasound of the supramammary lymph node in female sheep without lesion and low Somatic Cell Count; (B) Real-time ARFI elastography of the supramammary lymph node.

**Figure 2:**
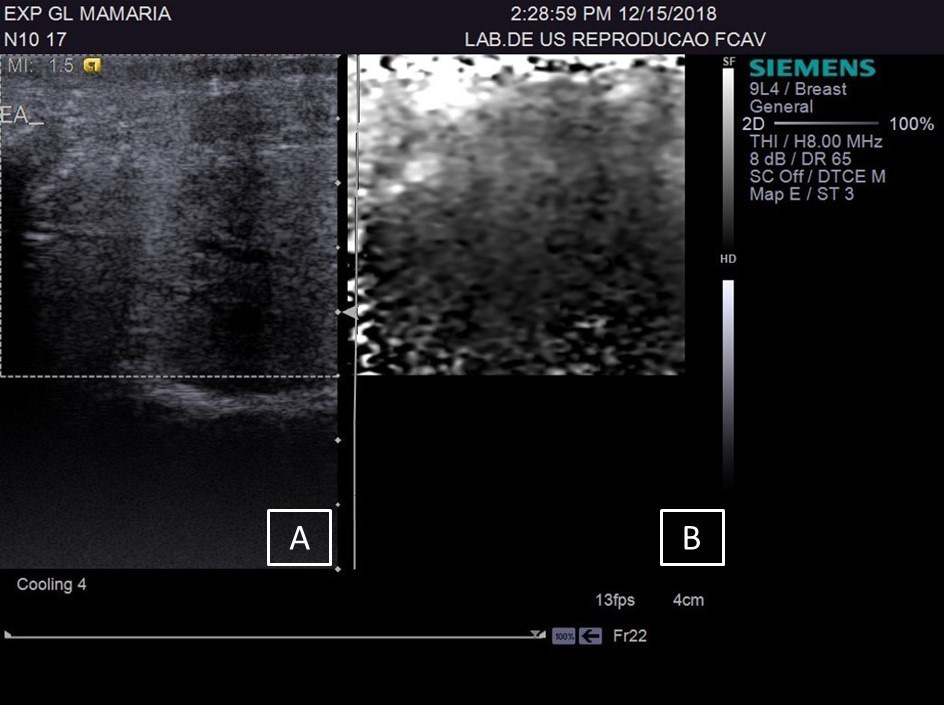
(A) B-mode ultrasound of the breast in female sheep without chronic lesions; (B) ARFI elastography in fibrous regions of the breast parenchyma, to obtain quantitative variables.

**Figure 3:**
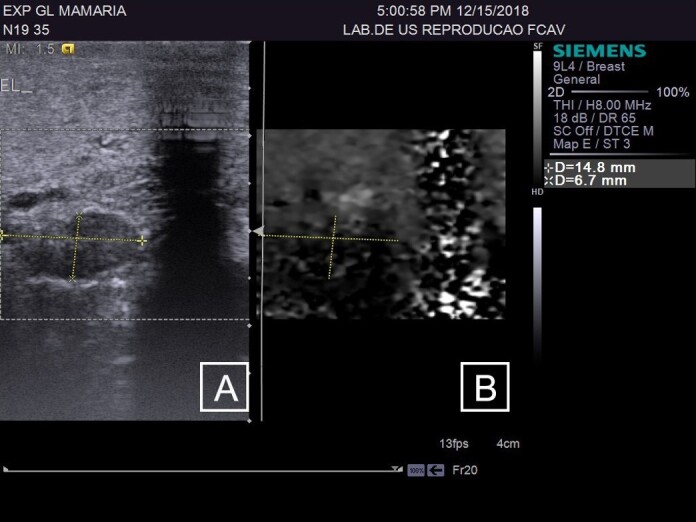
(A) B-mode ultrasound of the supramammary lymph node in female sheep with chronic lesions and high somatic cell count (HSCC); (B) Real-time ARFI elastography of the supramammary lymph node**.**

**Figure 4:**
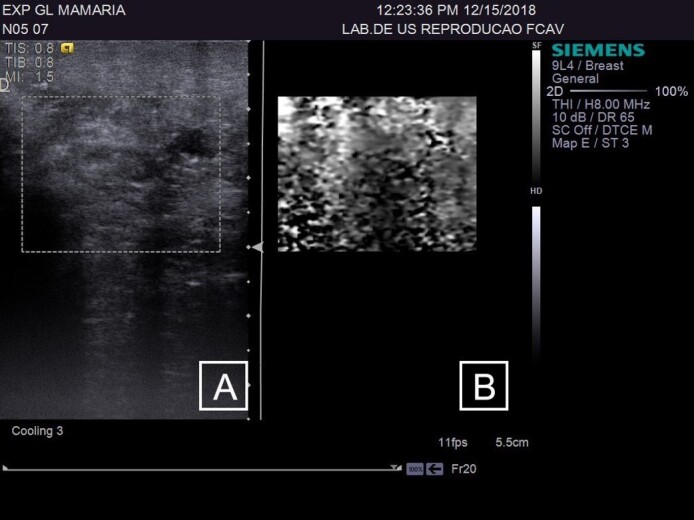
(A) B-mode ultrasound of the breast in female sheep with chronic lesions and high somatic cell count (HSCC), demonstrating areas of fibrosis, suggestive of chronic mastitis; (B) ARFI elastography in fibrous regions of the breast parenchyma, to obtain quantitative variables.

**Figure 5:**
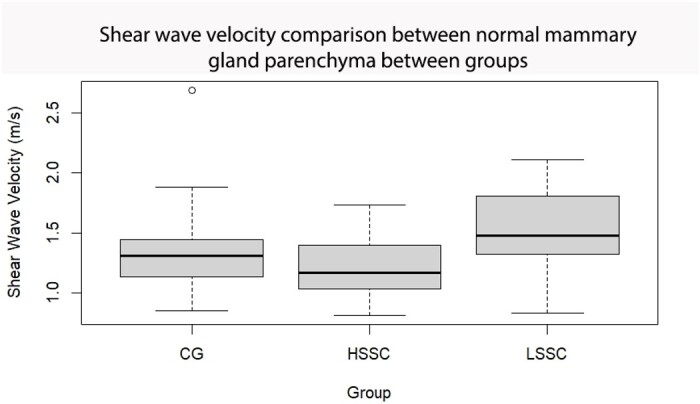
Variations in Shear Wave Velocity (mean ± SEM) as assessed by ARFI elastography across the mammary parenchyma of different sheep mamary gland groups: the control group (CG), normal parenchyma with fibrous lesions and a somatic cell count under 500 x 10^3^ cells/mL (LSCC), and normal parenchyma with a somatic cell count higher than 500 x 10^3^ cells/mL (HSCC). Notably, both the HSCC and LSCC groups exhibit fibrous lesions in health parts of the gland.

The CG group was composed of 19 mammary glands, the LSCC group included 18 glands, and the HSCC group comprised 11 glands. The sonographic assessments of the supramammary lymph nodes of GC (Figure 1) and breast parenchyma (Figure 2) was performed without any technical difficulties, as were those in the HSCC group (Figure 3 and Figure 4) and LSCC group. When comparing the normal breast parenchyma across all evaluated groups using quantitative ARFI, the mean SWV in LSCC was significantly higher than in HSCC (p=0.04). There was no significant difference in velocity between CG and HSCC (p=0.52; Figure 5). The depth in the CG was observed to be lower than in the LSCC and HSCC, although the differences did not reach statistical significance. The quantitative parameters of elastography and B-mode examination are detailed in Table 1.

**Table 1:** Description of the mean values ± standard deviation of the Somatic Cell Count (SCC) and quantitative variables, obtained by B-mode ultrasonography and ARFI elastography of three experimental groups, evaluating sheep of the Dorper x Santa Ines breed.

**TISSUE**	**GROUPS**	**N**	**QUANTITATIVE VARIABLES**			
			**SCC** **(x10^3^cel/ml)**	**Velocity** **(m/s)**	**Depth** **(cm)**	**Short/Long axis ratio**
Mammary gland	GC	19	144.2 ± 95.02	1.28±0.23^a-c^	0.76±0.16	-
	LSCC	18	122.06 ± 94.84	1.54±0.31^b^	0.94±0.25	-
	HSCC	11	3,980 ± 2,943.8	1.22±0.08^a^	0.87±0.31	-
Fibrous lesion	LSCC	18	-	2.18±0.59^d^	0.74±0.18	-
	HSCC	11	-	2.37±0.49^d^	0.70±0.12	-
Supramammary lymph node	GC	19	-	1.04±0.31^e^	1.62±0.64	0.41±0.08
	LSCC	18	-	1.25±0.41^e^	1.72±0.44	0.42±0.11
	HSCC	11	-	1.31±0.35^f^	1.64±0.61	0.38±0.08

^a,b^ - Equal letters in the same column, no difference between the groups, analysing the normal regions; ^c,d^ - Equal letters in the same column do not differ when comparing the means between the parenchyma of the CG group, and lesions in LSCC and LSCC, by the T-test (p<0.05); ^e, f^ - Equal letters in the same column do not differ when comparing the means between the CG, LSCC, and LSCC groups, by the T-test (p<0.05). N = number of mammary glands evaluated per group; LSCC = low somatic cell Count; HSCC = high somatic cell count; SCC = somatic cell counting.

The results revealed a significant increase in shear velocity in the lesions compared to the normal regions of the gland in both the LSCC group (p=0.001) and the HSCC group (p=0.0001).

**Figure 6:**
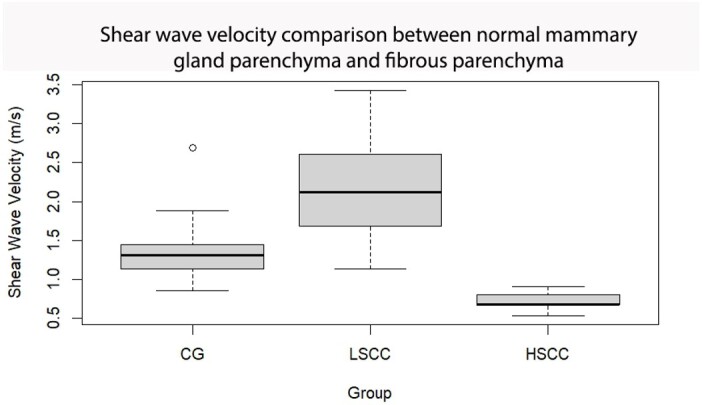
Comparison of Shear Wave Velocity (mean ± SEM) in the normal parenchyma of the control group (CG) with fibrous lesions in the LSCC group (somatic cell count under 500 x 10^3^ cells/mL) and the HSCC group (somatic cell count higher than 500 x 10^3^ cells/mL), of the mammary gland in sheep, with or without mastitis.

When comparing the velocity in lesions between the LSCC and HSCC groups at Tukey-test, it was higher in the first group (p < 0.001, approximately 1.55 x 10^^-9^). However, when comparing the lesions with the healthy CG group, there was a significant increase in velocity in LSCC (p < 0.001, approximately 5.42 x 10^^-6^) and HSCC (p= 0.00261; Figure 6). The lesions compared between LSCC and HSCC was higher in the first group (p < 0.001, approximately 1.55 x 10^^-9^).

**Figure 7:**
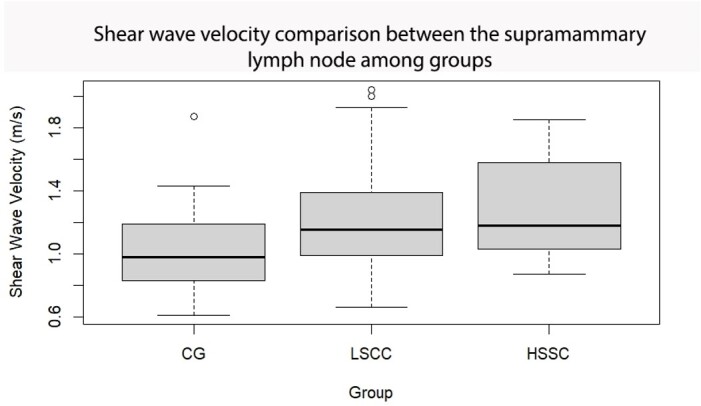
Shear Wave Velocity (mean ± SEM) in the supramammary lymph nodes of study groups: control group (CG), fibrous lesions at parenchyma with somatic cell count under 500 x 10^3^ cells/mL (LSCC), and fibrous lesions with somatic cell count higher than 500 x 10^3^ cells/mL (HSCC) in sheep with or without mastitis.

Analysing the SWV of the supramammary lymph nodes in each gland and comparing the groups, a significant difference was observed between the CG and HSCC groups (p=0.04), and CG and HSCC (p=0.02) with the latter group exhibiting higher velocities (Figure 7). The ratio between the short and long axis of the supramammary lymph nodes did not show a significant difference between groups, but the predominant shape was elongated in all groups. The qualitative variables observed during B-mode ultrasonography, including echotexture (p=0.9336) and echogenicity (p=0.233), showed no significant differences between the evaluated groups and tissues, with no correlation observed between these variables. Similarly, there was no correlation observed when evaluating the qualitative variables of ARFI elastography, including deformation capacity (p=0.29) and pattern (p=0.7). The corresponding data have been presented in Table 2.

**Table 2:** Qualitative variables, presented according to the proportion in each group, obtained from B-mode ultrasonography and ARFI elastography of healthy mammary glands, fibrous lesions and supramamammary lymph nodes, in sheep of the different experimental groups.

**Tissue**	**Group**	**Qualitative B-mode Variables**				**ARFI Qualitative Variables**			
		**Echogenicity (Ratio %)**		**Echotexture (Ratio %)**		**Deformation (Ratio %)**		**Standard (Ratio %)**	
Mammary gland	GC	Hippo	89.5	Homo	5.3	Hard	100	Homo	94.7
		Hyper	10.5	Hetero	94.7	Soft	0	Hetero	5.3
		Mixed	0						
	LSCC	Hippo	100	Homo	0	Hard	100	Homo	83.3
		Hyper	0	Hetero	100	Soft	0	Hetero	16.7
		Mixed	0						
	HSCC	Hippo	81.8	Homo	0	Hard	90.9	Homo	100
		Hyper	9.2	Hetero	100	Soft	9.1	Hetero	0
		Mixed	0						
Fibrous Lesion	LSCC	Hippo	88.9	Homo	5.6	Hard	100	Homo	0
		Hyper	11.1	Hetero	94.4	Soft	0	Hetero	100
		Mixed	0						
	HSCC	Hippo	90.9	Homo	0	Hard	100	Homo	0
		Hyper	9.1	Hetero	100	Soft	0	Hetero	100
		Mixed	0						
Supramammary lymph node	GC	Hippo	100	Homo	95.4	Hard	50	Homo	95.4
		Hyper	0	Hetero	5.6	Soft	50	Hetero	5.6
		Mixed	0						
	LSCC	Hippo	100	Homo	95.4	Hard	66.67	Homo	95.4
		Hyper	0	Hetero	5.6	Soft	33.33	Hetero	5.56
		Mixed	0						
	HSCC	Hippo	100	Homo	81.8	Hard	81.8	Homo	100
		Hyper	0	Hetero	18.2	Soft	18.2	Hetero	0
		Mixed	0						

Hypo - hypoechoic; Hyper - hyperechoic; Homo - homogeneous; Hetero – heterogeneous.

## Discussion

B-mode ultrasonography has demonstrated its effectiveness in evaluating the components of the mammary gland in sheep, as previously reported by Hussein et al. (2015) and Barbagianni et al. (2017). This effectiveness was further confirmed in the present study, where the structures were easily identified during the evaluation of the female subjects. It is worth mentioning that previous studies described the positioning of animals and the technique performed in a quadrupedal position (Hussein et al., 2015; Barbagianni et al., 2017). In our study, however, we maintained the animals with the elevation of thoracic limbs and contact of the gluteal muscles with the ground during the examinations. This positioning facilitated proper restraint and allowed comprehensive evaluation of the entire mammary apparatus, ensuring the safety of both the evaluator and the animals involved,

B-mode ultrasonography has been recognized as an effective method for evaluating supramammary lymph nodes in sheep (Hussein et al., 2015), similar to what has been observed in cattle (Bradley et al., 2001); and these lymph nodes play a crucial role in the defense mechanism of the mammary gland (Watson and Davies, 1985). In our study, however, the qualitative aspects of the supramammary lymph nodes, including echogenicity and echotexture, did not showed significant differences among the three experimental groups, but a predominance of hypoechoic echogenicity was observed in the GC, LSCC, and HSCC groups, with only a few anechoic points.

These findings are consistent with previous studies in cattle conducted by Bradley et al. (2001) and in sheep with subclinical mastitis reported by Hussein et al. (2015). In addition to echogenicity and echotexture, the tissue elasticity pattern was assessed, and a rigid pattern was predominantly observed in LSCC (66.67%), which further increased in HSCC (81.88%). These results indicate that the lymph nodes become increasingly rigid in the presence of fibrosis and mastitis. Overall, these findings support the notion that B-mode ultrasonography is a valuable tool for assessing the supramammary lymph nodes in sheep, providing insights into their response to infectious processes such as mastitis.

The quantitative analysis of ARFI elastography in the mammary parenchyma revealed more significant results compared to the qualitative analysis. In addition to assessing the fibrous regions in LSCC and HSCC, measurements were taken in healthy areas of the glands in these groups to identify potential changes in relation to normal glands (CG) and those with subclinical mastitis (HSCC).

The analysis of the normal tissue demonstrated that the SWV in LSCC was higher compared to the other groups. The increased stiffness suggests that the chronic process of lesions caused by microorganisms, which lead to the formation of hard and firm nodules (Marogna et al., 2010), influences the elastic properties of the entire mammary parenchyma. This finding is supported by a study on human pancreas, where higher SWV were observed in chronic pancreatitis, and lower values were found in acute pancreatitis (Goertz et al., 2016). Previous studies using ARFI elastography have also observed that mean velocity in subcutaneous adipose tissue was lower than in breast parenchyma, and mean velocity of malignant lesions was significantly higher than in benign lesions (Tozaki et al. 2011). These findings indicate that after the active inflammatory process of mastitis, the normal mammary parenchyma in ewes with areas of fibrosis may exhibit increased stiffness, independent of focal fibrosis formation. This phenomenon was not observed in ewes without fibrosis (GC), suggesting that fibrosis development in the mammary gland affects the overall elasticity of the tissue. However, it is important to note that there was no evidence of previous mastitis presence in the females of the control group, which limits the ability to obtain more conclusive results. Nonetheless, the low shear velocities observed in the parenchyma without fibrosis and with low somatic cell count (CG) may suggest that these glands are free from mastitis.

When comparing the normal breast parenchyma regions of LSCC and HSCC with their respective peripheral fibrous lesions, the significant increase in shear velocities demonstrated the potential of ARFI elastography for differentiating fibrous areas, indicative of chronic lesions. Similar findings have been observed in women, where increased breast parenchymal stiffness is associated with idiopathic granulomatous mastitis and invasive ductal carcinoma (Toprak et al., 2022), as well as in female dogs, where mammary tumours exhibit higher velocities (Feliciano et al., 2017). This increase in stiffness may be attributed to inflammatory changes resulting from bacterial invasion into the mammary parenchyma and subsequent fibrosis (Addis et al., 2013).

The comparison of shear velocities in fibrosed areas of LSCC and HSCC with the normal parenchyma of GC revealed a significant increase in shear velocities in LSCC and CG compared to HSSC. This indicates that ARFI elastography shows higher velocities in LSCC, followed by CG, and lower velocities in HSSC. It is important to highlight that although this observation suggests promising results, it had not been previously documented before this study. This implies that evaluating fibrosed areas could potentially enhance the identification of old chronic lesions without mastitis as active mastitis with chronic lesions. However, it is important to note that assessing fibrosis alone may not be a reliable parameter for differentiating active from chronic mastitis

ARFI elastography has demonstrated its efficacy in lymph node analysis, as evidenced by studies conducted on human axillary lymph nodes for differentiating malignant from benign breast tumours, showing increased SWV in malignant cases (Youk et al., 2017). This finding has also been replicated in female dogs, where increased SWV was observed in acute inflammatory processes compared to normal lymph nodes (Silva et al., 2018).

In our study, the quantitative analysis of SWV between the supramamammary lymph nodes of the GC, LSCC, and HSCC groups revealed a significant increase in the HSCC group compared to GC, and a smaller increase in LSCC compared to GC. This increase in the group with active mastitis could be attributed to the enhanced production and accumulation of leukocytes within these lymph nodes (Watson and Davies, 1985). This finding is supported by studies conducted in rats, where a positive correlation was observed between the abundance of CD3-T lymphocytes in the liver of obese rats and shear rate (Carbonell et al., 2019).

Similarly, in sheep, a higher distribution of T lymphocytes has been observed in the cortex of supramamammary lymph nodes (Senthilkumar et al., 2019), and their increase in response to mammary gland infection (Katsafadou et al., 2019), which could contribute to the elevated SWV observed in HSCC. The interpretation of these results suggests that increased SWV in the supramamammary lymph nodes of glands with fibrosis in the parenchyma, along with high SCC, may serve as a useful diagnostic tool for active subclinical mastitis. However, further studies are required to solidify this hypothesis and differentiate it from the response of normal mammary parenchyma.

There is limited research available regarding the evaluation of supramammary lymph node depth in sheep for the diagnosis of subclinical mastitis. A single study by Hussein et al. (2015) describes depths greater than 7.8 mm as characteristic of sheep with subclinical mastitis. In contrast, studies conducted on cows have shown varying results, with some reporting reduced lymph node depth in subclinical mastitis (Khoramian et al., 2015) and others reporting increased depth (Bradley et al., 2001).

In our study, the mean value for supramamammary lymph node depth in the HSCC group was 16.4 mm (1.64 cm), with no significant difference observed between the other groups. These findings differ from the values reported for sheep with subclinical mastitis in the study by Hussein et al. (2015). It is important to note that the interpretation of lymph node depth as a diagnostic parameter for subclinical mastitis may vary depending on the species, and further research is needed to establish consistent and reliable guidelines for the interpretation of lymph node depth in different animal populations.

In addition to evaluating the echogenicity, echotexture, stiffness, and quantitative ARFI of the lymph nodes, we also measured the short axis (width) and long axis (length) to calculate the short axis/long axis ratio. The results showed lower ratios in the HSCC group, followed by higher ratios in the LSCC and CG groups. These findings are consistent with previous studies in sheep (Hussein et al., 2015) and cattle (Bradley et al., 2001), where an increase in the short axis was observed. The predominant shape of the evaluated lymph nodes in all groups was elongated, which aligns with the findings of Hussein et al. (2015). However, there is a tendency for the lymph nodes in subclinical mastitis with fibrosis in the mammary parenchyma to become more circular, as indicated by an increased short axis. This observation has not yet been reported in ruminants and represents a novel finding. This information is significant as it provides additional insights into the evaluation of the mammary gland in this species and lays the groundwork for future studies.

## Conclusion

B-mode ultrasonography is an excellent tool for evaluating the mammary parenchyma, facilitating the detection of fibrosis resulting from infectious mastitis and identifying supramammary lymph nodes. However, its application as a standalone diagnostic technique for subclinical mastitis in sheep is not recommended. Sonographic findings should be compared with complementary tests for accurate diagnosis. Additionally, a decrease in the short/long axis ratio and an oval shape of the lymph nodes may indicate active mastitis, particularly noteworthy given the presence of previous fibrous lesions in the sheep.

ARFI elastography showed different velocities in fibrosed areas compared to normal parenchyma, indicating its effectiveness in diagnosing fibrosis., suggesting thatit may assist in differentiating active from chronic mastitis. However, we advise that further studies with a larger sample size and controlled assessment of fibrosis severity are necessary to potentially establish cutoff values to differentiate subclinical mastitis processes from fibrous gland conditions. Promising results are observed in the evaluation of supramammary lymph nodes, where high SWV and increased stiffness may indicate subclinical mastitis.
